# A ‘living fossil’ eel (Anguilliformes: Protanguillidae, fam. nov.) from an undersea cave in Palau

**DOI:** 10.1098/rspb.2011.1289

**Published:** 2011-08-17

**Authors:** G. David Johnson, Hitoshi Ida, Jiro Sakaue, Tetsuya Sado, Takashi Asahida, Masaki Miya

**Affiliations:** 1 Division of Fishes, National Museum of Natural History, Smithsonian Institution, Washington, DC 20560, USA; 2 School of Marine Biosciences, Kitasato University, Ofunato, Iwate 022-0101, Japan; 3 Southern Marine Laboratory, PO Box 1598, Koror 96940, Palau; 4 Natural History Museum and Institute, Chiba, Chuo-ku, Chiba 260-8682, Japan

**Keywords:** eel, morphology, phylogeny, new species, genus and family, divergence time

## Abstract

We report the discovery of an enigmatic, small eel-like fish from a 35 m-deep fringing-reef cave in the western Pacific Ocean Republic of Palau that exhibits an unusual suite of morphological characters. Many of these uniquely characterize the Recent members of the 19 families comprising the elopomorph order Anguilliformes, the true eels. Others are found among anguilliforms only in the Cretaceous fossils, and still others are primitive with respect to both Recent and fossil eels. Thus, morphological evidence explicitly places it as the most basal lineage (i.e. the sister group of extant anguilliforms). Phylogenetic analysis and divergence time estimation based on whole mitogenome sequences from various actinopterygians, including representatives of all eel families, demonstrate that this fish represents one of the most basal, independent lineages of the true eels, with a long evolutionary history comparable to that of the entire Anguilliformes (approx. 200 Myr). Such a long, independent evolutionary history dating back to the early Mesozoic and a retention of primitive morphological features (e.g. the presence of a premaxilla, metapterygoid, free symplectic, gill rakers, pseudobranch and distinct caudal fin rays) warrant recognition of this species as a ‘living fossil’ of the true eels, herein described as *Protanguilla palau* genus et species nov. in the new family Protanguillidae.

## 1. Introduction

Ever since Charles Darwin coined the term ‘living fossil’ in *On the Origin of Species* (p. 107 in [1]), organisms that have been called living fossils have received considerable attention. These extremely long-lived or geologically long-ranging taxa with few morphological changes can aid in forming a picture of ancient forms of life. Most ancient forms of life, however, have gone extinct with no known fossil remnants. Exceptions are represented by a few extant animal lineages that have remained morphologically static over geological time scales (e.g. horseshoe crabs, plethodontid salamanders and lampreys [2]).

**Figure 1.**
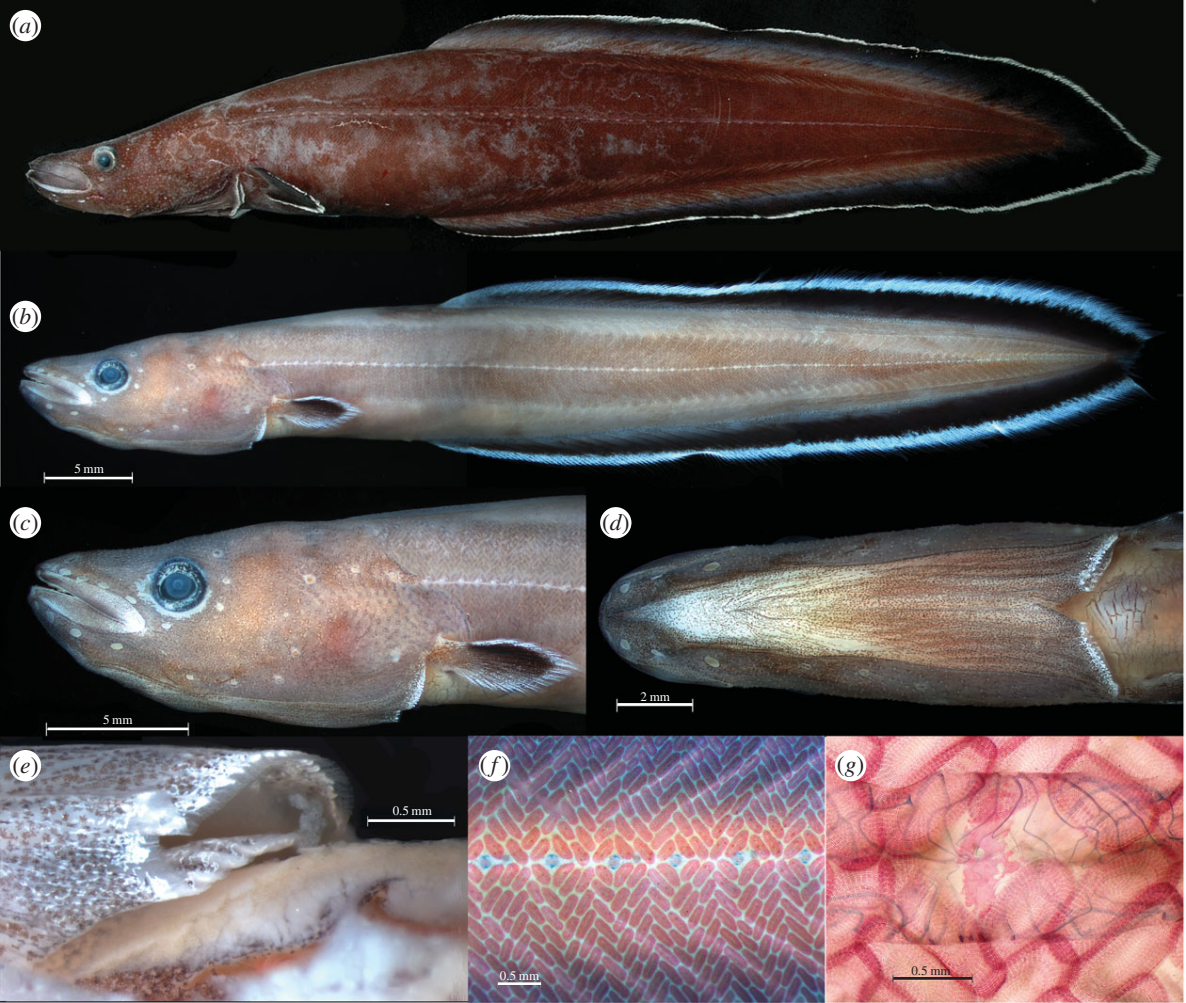
*Protanguilla palau*. (*a*) Holotype, NSMT-P 98249 female, 176 mm SL. (*b–g*) Paratype USNM 396016 juvenile, 65 mm SL: (*b*) whole specimen; (*c,d*) head in lateral and ventral view, respectively; (*e*) close-up of tubular gill opening, left side in ventral view; (*f*) alizarin red-stained body scales along lateral midline (lateral-line scales are forming in alcian blue-stained areas); (*g*) USNM 396051, 150 mm SL, alizarin red-stained, close-up of lace-like, tubular lateral-line scale.

Recently one of us (J.S.) collected a small eel-like fish from a 35 m-deep fringing-reef cave in Palau. Compared with true eels, this fish has a disproportionately large head, short compressed body, distinctive collar-like gill openings and slightly produced caudal fin rays (figure 1*a*–*e*; see also video in the electronic supplementary material). Despite some early questions about its affinities, preliminary phylogenetic analysis based on whole mitogenome sequences and numerous osteological features confidently placed this fish within the true eels. Additional morphological and molecular analyses demonstrate that in some features it is more primitive than Recent eels, and in others, even more primitive than the oldest known fossil eels, suggesting that it represents a ‘living fossil’ without a known fossil record.

Anguilliformes are a distinctive group of teleosts, comprising 819 species in 19 families and 146 genera [3,4]. They share a unique ribbon-like ‘leptocephalus’ larva [4] with their closest relatives, tarpons (Order Elopiformes), bonefishes (Order Albuliformes) and notacanths (Order Notacanthiformes) [5,6]. Anguilliforms first appeared as fossils in the Cretaceous about 100 million years ago (Ma) [7] and have lost their pelvic fins, and their dorsal, anal and caudal fins have become confluent [8]. Many eels are adapted for occupying small spaces or burrowing, but they occur in diverse habitats, ranging from benthic shallow-water to deep-shelf, slope and abyssal plain, open-water, meso- and bathypelagic realms [4].

Here, we describe a new family, genus and species for this enigmatic eel. We demonstrate, based on convincing evidence from morphology and whole mitochondrial genomes, that this genus is the most primitive living member of the Anguilliformes, and we accordingly assign it to a new family. In accordance with article 8.6 of the International Code of Zoological Nomenclature, copies of the PDF file of this work have been deposited in the following five publicly accessible libraries: (i) National Museum of Natural History, Smithsonian Institution, Washington, DC; (ii) Natural History Museum, London; (iii) National Museum of Nature and Science, Tokyo; (iv) Field Museum, Chicago; and (v) American Museum of Natural History, New York.

## 2. Material and methods

### (a) Morphology

Counts and measurements of all eight known specimens, follow Böhlke [9]. All sizes in millimetres are standard length. Institutional abbreviations are as listed at http://www.asih.org/codons.pdf (see the electronic supplementary material for further details and comparative specimens).

### (b) Molecular methods

A whole mitogenome sequence from one of the specimens (CBM-ZF 12278) was determined using a combination of long and short polymerase chain reactions (PCRs) and direct cycle sequencing techniques [10].

Mitogenome sequences from the new eel and various actinopterygians were concatenated with the pre-aligned sequences used by Azuma *et al*. [11] with MAFFT v. 6 [12]. To address issues of the phylogenetic positions of the new eel (i) among Actinopterygii and (ii) within Anguilliformes, and (iii) to investigate its divergence time, we constructed three datasets based on different taxon and character sampling (electronic supplementary material, table S3). Character sampling in datasets 1 and 3 follows Azuma *et al*. [11], who excluded entire third codon positions from protein-coding genes because of their positively misleading phylogenetic signal at higher taxonomic levels [13,14] and because of their extremely accelerated rates of changes, which may overestimate divergence times [15]. For dataset 2, we added transversional changes in the third codon positions for resolving relationships within the order, following Inoue *et al*. [3]. Unambiguously aligned sequences were divided into four or five partitions (two or three codon positions, rRNA and tRNA genes) and subjected to partitioned maximum-likelihood (ML) analysis using RAxML v. 7.2.6 [16]. The best-scoring ML tree was estimated using a general time reversible (GTR) + *Γ* model of sequence evolution with 1000 bootstrap replicates. Probabilities of alternative hypotheses were calculated using the likelihood-based approximately unbiased (AU) test as implemented in CONSEL v. 0.1k [17].

A relaxed molecular-clock method implemented in an MCMCTREE program in PAML v. 4.4 [18] was used for dating analysis. One of the constrained, best-scoring ML trees that are congruent with the morphology-based phylogenetic placement of the new eel was used for divergence time estimation (see below). The ML estimates of branch lengths were obtained under the GTR + *Γ* substitution model. The independent-rates (IR) model was used to specify prior of rates among internal nodes. Twelve fossil-based time constraints from Azuma *et al*. [11] were used (electronic supplementary material, table S4).

More details of the methods can be found in the electronic supplementary material.

## 3. Results and discussion

### (a) Taxonomy

**Figure 2.**
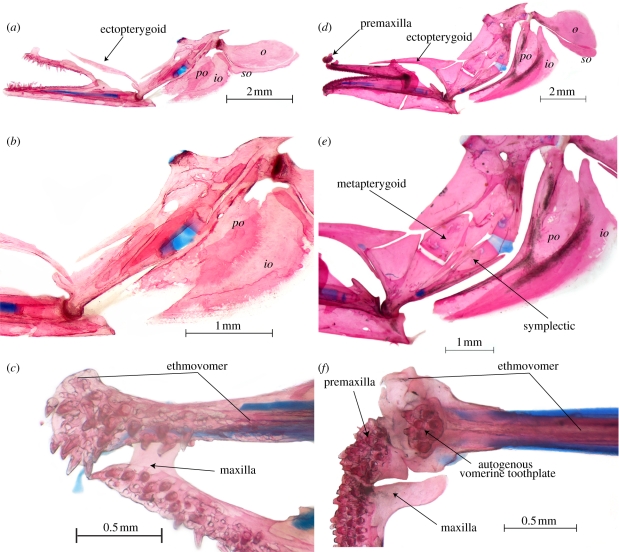
(*a–c*) *Anguilla rostrata*, USNM 106563, 91 mm SL. (*a,b*) Suspensorium and opercular series, left lateral view and close-up, respectively; (*c*) ethmovomer and upper jaw, ventral view. (*d–f*) *Protanguilla palau*, USNM 396016, 65 mm SL. (*d,e*) Suspensorium and opercular series, left lateral view and close-up, respectively; (*f*) ethmovomer and upper jaws, ventral view. *io*, interopercle; *o*, opercle; *po*, preopercle; *so*, subopercle.

**Figure 3.**
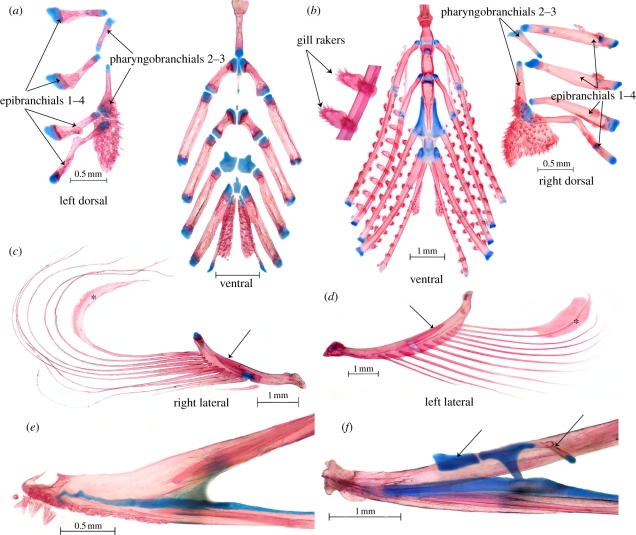
(*a,c,e*) *Anguilla rostrata*, USNM 106563, 91 mm SL; (*b,d,f*) *P. palau*, USNM 396016, 65 mm SL. (*a,b*) Gill arches, dorsal view; note presence of gill rakers in (*b*). (*c,d*) Hyoid arch with branchiostegal rays; note spatulate last branchiostegal rays (asterisks), absence of interhyal and autogenous hypohyals, and posterior extension of anterior ceratohyal over dorsal edge of posterior ceratohyal (arrows). (*e,f*) Ethmovomer, left lateral view; arrows point to perichondral ossification of lateral ethmoid and separate anterior cartilage.

**Figure 4.**
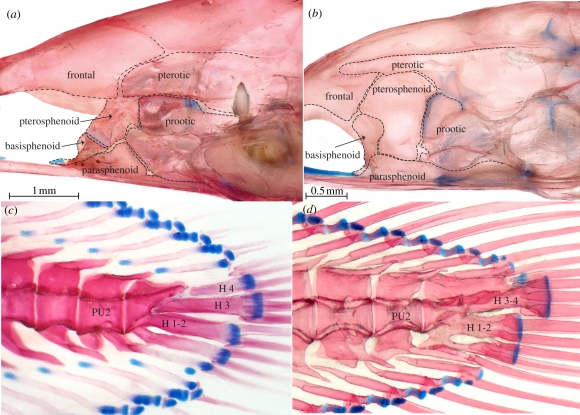
(*a–b*) Mid-portion of braincase of (*a*) *P. palau*, USNM 396016, 65 mm SL, and (*b*) *S. parasiticus*, USNM 326917, 78 mm SL. (*c–d*) Caudal skeleton of (*c*) *P. palau*, USNM 396016, 65 mm SL, and (*d*) *A. rostrata*, USNM 106563, 91 mm SL. PU2*,* preural centrum 2; H*,* hypural.

(i) Protanguillidae (Palauan primitive cave eels; Ngkelelaruchel; Mukashi-unagi) fam. nov. Johnson, Ida & Miya (figures 1–4).

Diagnosis: Gill opening terminates as ovoid tube with low, fringed collar; pseudobranch present; knob-like, toothed gill rakers present; premaxilla present, autogenous; symplectic autogenous; metapterygoid present; anterior end of vomer with small, ovoid, autogenous toothplate; body relatively short, total vertebrae 87 or fewer (79–87, mean = 83.3); hypurals 3 and 4 not fused to each other; pterosphenoid not excluded from posterior margin of orbit.

(ii) *Protanguilla* gen. nov. Johnson, Ida & Miya.

Diagnosis: That of the family.

Type species: *Protanguilla palau* sp. nov.

Etymology: From the Greek *protos*, first, and the Latin *anguilla*, eel, in reference to the early divergence of the genus among anguilliforms.

(iii) *Protanguilla palau* sp. nov. Johnson, Ida & Sakaue.

Holotype: NSMT-P 98249, female (176 mm SL), cave at 35 m depth, western fringing reef of Ngemelis Island, Republic of Palau, collected with hand net and torch light by J. Sakaue, 30 March 2009.

Paratypes: Collected from same location as holotype: FSKU-P24231, 58.2 mm SL, 16 November 2009; FSKU-P24232, 43.9 mm SL, 17 February 2009; USNM 396016, 2 (60, 65.2 mm SL), 16 November 2009; USNM 396051, 150 mm SL, 30 March 2009; USNM 396052, 45.5 mm SL, 24 April 2010; NSMT-P 98250, 46.3 mm SL, 17 February 2009; CBM-ZF 12278, 49.6 mm SL, 17 February 2009; CBM-ZF 12279, 71 mm SL, 16 November 2009.

Description: Body elongate, snout depressed; opercular region sub-cylindrical, mid-trunk moderately laterally compressed, posterior portion of tail extremely laterally compressed; pelvic fins absent; pectoral fins inserting on lower 1/3 of body; dorsal and anal fin bases long, exceeding 2/3 body length. Gill membranes united, fused with isthmus. Scales minute, elliptical, embedded; arranged in basket weave pattern on body, absent from around eye, lips and anterior part of snout; those on basal part of median fins and lower jaw ovoid; lateral-line scales formed by tubular latticework of flexible bone extending outward from ovoid basal plate. All fin rays bilaterally paired and segmented, only those of pectoral branched; pectoral fin rays 19 (18–19 in paratypes); dorsal fin rays 182 (176–189); anal fin rays 181 (175–191); caudal fin rays 10 (5 + 5); vertebrae 21 preanal + 66 post-anal (20–23 + 56–64); lateral-line scales 80 (80–84). Neural spines well developed on all vertebrae, anterior approximately 16 with broad laminar expansions that enclose yellowish fat globules; neural arches forming a tunnel-like shield around spinal cord and firmly interlocked with adjacent arches. Lateralis system (terminology after Böhlke [9]) on head conspicuous, openings large with elevated fringe; m 4, pop 4, io 7, so 4, e 1, T 2; lateral line complete. Knob-like, toothed gill rakers present in two rows on each arch; 0–4 on outer row of first upper arch, 9–15 on outer row of first lower arch (variation ontogenetic). Jaw and pharyngeal teeth villiform. Upper and lower lips well developed, thick. Pyloric caeca absent. Sagitta extremely large, length more than 1/4 head length. Anterior nostril opens in short tube just above upper lip, posterior just before anterior rim of orbit without tube. Olfactory rosette large, about one-third snout length, lamellae approximately 40.

Etymology: Palau, where the type series was collected, a noun in apposition.

### (b) Morphological evidence

Robins [8] listed 38 ‘general features’ of Recent adult eels, noting that ‘most are not specializations (synapomorphies) of the order, but are shared with other primitive fishes’ (p. 13 in [8]). He also concluded that the Cretaceous genera *Anguillavus*, *Urenchelys* and *Enchelion* are not eels, a conclusion later rejected [19] and conclusively refuted by two more recent, detailed morphological and phylogenetic doctoral theses [20,21], and two follow-up papers [7,22] that erected three additional genera (*Luenchelys*, *Hayenchelys* and *Abasaadia*), based on cladistic analyses. Two other Cretaceous taxa are excluded from consideration here: *Eoenchelys*, because of its placement as the sister group of the highly derived Saccopharyngoidei; and *Enchelurus*, because of its putative placement as the sister group of Anguilliformes plus Notacanthiformes [20]. We also exclude the latter group from comparative discussion, because of their further specializations (e.g. extreme reduction of the entire caudal region [23]).

To date, there is no morphology-based consensus on which Recent eels are the most primitive. Conjectures based on gill-arch structure [24] and other osteological features [25–27] suggest that they would most probably be found among the families Synaphobranchidae, Congridae or Anguillidae, and the results of the fossil-inclusive analyses [20,22] generally concur.

We have critically reviewed characters of Recent and Cretaceous eels proposed in previous studies (e.g. [7,20,22,28–30]) and additional features, and herein identify characters that cladistically diagnose the Recent eels and variously fossil-inclusive groups, based on outgroup comparison with basal elopomorphs [3,6,29].

The following morphological features are those shared by *Protanguilla* and all anguilliforms (including the Cretaceous *Abisaadia*, *Luenchelys*, *Anguillavus*, *Hayenchelys* and *Urenchelys*) that we find to be unique or rare enough at this level to be considered synapomorphic for the order.

— Ethmoid fused with vomer (figure 3). In all Recent and fossil eels, the snout is formed by a single bone that bears teeth, meets the parasphenoid ventrally, and meets the frontals and encloses the ethmoid commissural sensory canal (when present) dorsally [8,20]. This bone is generally called the ethmovomer, and has been shown to incorporate the ethmoid and vomer, which fuse in early ontogeny [31]. Reports of a separate vomer in some eels (e.g. *Simenchelys* [32]; *Pythonichthys* [33]; *Luenchelys* [30]) are erroneous, as this bone can easily be seen to be an autogenous toothplate underlying the true vomer.
— Pterotic extends anteriorly above prootic to contact pterosphenoid [7,20,28,29] (figure 4).
— Dermopalatine and autopalatine absent [33] (figure 2). There is no morphological or ontogenetic support for the surmise [8,34] that the palatine is fused into the ethmovomer in many eels. The autopalatine ossifies in the palatine process of the palatoquadrate and, as far as is known [31,35–37], this fails to develop in eels. The ‘palatine’ described and illustrated as separate in *Serrivomer* [38–40] is clearly a dermal element, described elsewhere as fused to the pterygoid [41]. If there is a separate bone in serrivomerids, the condition must be secondary [8].
— Pectoral girdle displaced posteriorly [8,20], so that the junction of the supracleithrum with the cleithrum is at or posterior to the fourth vertebra.
— First pharyngobranchial absent and pharyngobranchials without uncinate processes [24] (figure 3). Pb1 is present, but unossified, only in chlopsids (except *Chilorhinus*) and the congrids *Pseudophichthys* and *Ariosoma*. Unreported for fossils.
— Gill arches free from braincase and displaced posteriorly [8,20,29]. Among Recent eels, only *Simenchelys* and *Protanguilla* have the first two epibranchials located anterior to the occiput, a position similar to that in Cretaceous forms, except *Luenchelys*, in which the position is as in other Recent eels [30].
— Opercular series characterized by a distinctive pattern in which the opercle is rostrocaudally elongated with a bottle-neck articular condyle and broadly bordered ventrally by subopercle [20,41] (figure 2).
— Uppermost branchiostegals curving dorsally behind and often slightly above opercle [8,20,42] (figure 2).
— Posterior ceratohyal almost equal to or longer than anterior ceratohyal (figure 3). While it is true that the ‘anterior end of the ceratohyal is elongated’ (p. 11 in [8]), we find that the unusual feature of the hyoid bar of most eels, including Cretaceous forms [20,21,30], compared with that of other teleosts, is elongation of the posterior ceratohyal relative to the anterior ceratohyal. Notable exceptions are found in *Derichthys* and some ophichthids [43].
— Branchiostegals more numerous on the posterior than on the anterior ceratohyals [20,42] (figure 3; equally distributed in *Luenchelys* [7,30]).
— Posteriormost one to four branchiostegals with spatulate expansions distally (figure 3). This expansion is well developed among Recent eels in anguillids, synaphobranchids, heterenchelyids, moringuids and *Protanguilla*, and its widespread occurrence in Cretaceous forms leads us to conclude that it is a synapomorphy of eels [7,20,42].
— Dorsal part of suture between anterior and posterior ceratohyals deflected posteriorly (figure 3). In Recent [41] and Cretaceous [20] eels, with the possible exception of *Luenchelys* [30], the anterior ceratohyal sends back a dorsal projection to overlie the dorsal edge of the posterior ceratohyal.
— Interhyal absent in adults [42] (figure 3). In Recent eels, the interhyal appears in cartilage early in development, never ossifies, and is lost in the post-metamorphic glass eel stage [31,36,43]. In adults of Recent eels that we examined, the posterior ceratohyal attaches by a cord-like ligament, above the usual articulation site of the interhyal, to the medial face of the hyomandibular. An interhyal has not been reported in Cretaceous eels [20,30].
— Angular, articular and retroarticular fused into a single bone [20,29,44] ontogenetically [31] (figure 2).
— Two pairs of upper pharyngeal toothplates present and autogenous (not fused to pharyngobranchials); lower pharyngeal toothplates autogenous (not fused to fifth ceratobranchials) except in some species of *Conger* [24] (figure 3). Data for Cretaceous forms are limited.

The following are synapomorphies of Recent eels and *Protanguilla* lacking in Cretaceous eels:

— Endopterygoid absent (figure 2). The palate of all Recent eels comprises a single dermal bone [8], the ectopterygoid. Cretaceous eels also have a dermal endopterygoid [7,20,22].
— Scales on body absent (or, when present, non-imbricate), embedded and arranged in ‘basket-weave fashion’ [8] (figure 1). A similar (though distinguishable) pattern is found in some zoarcoids and ophidioids. Most anguilliforms lack scales, but they are present in this pattern in *Anguilla*, synaphobranchids and *Protanguilla*. Cycloid, non-imbricate scales have been reported in some Cretaceous eels, but not in the distinctive basket-weave pattern [21,45]. Another unusual feature of *Protanguilla* is the presence of lace-like lateral-line scales, also found among ophichthid, congrid and nettastomatid eels, all of which lack body scales [46]. Tubular lateral line scales have also been reported in some Cretaceous forms [21,37], but whether solid or lace-like is unclear.
— One or no hypohyals (figure 3). In most Recent eels, the anterior end of the hyoid bar is fully ossified, with no separate hypohyal [42]. A single hypohyal occurs in *Myroconger*, *Coloconger*, serrivomerids, and at least some chlopsids, synaphobranchids, ophichthids, muraenesocids, nemichthyids and perhaps congrids. All other Recent eels examined have no separate hypohyal. The only Recent eel with two hypohyals is the synaphobranchid *Simenchelys* [32], but two are found in the Cretaceous forms [7,20].
— Dorsal and anal fins confluent with caudal fin (figure 4). All Recent eels have the dorsal and anal fins confluent with the caudal [8,20,47], but in *Simenchelys* [8,32,47] there is a gap between the last dorsal pterygiophore and fin ray and the caudal skeleton and uppermost caudal fin ray. A similar gap is seen dorsally and ventrally in *Protanguilla*, and the caudal fin rays are abruptly longer than the adjacent dorsal and anal fin rays. Among Cretaceous eels, the last dorsal and anal pterygiophores are well anterior to the neural and haemal spines of the second preural centrum, the most posterior being those of *Urenchelys* (posterior to second and third preural centra, respectively) [7,20].
— Caudal fin rays fewer than eight in each lobe (figure 4). Among Recent eels, only *Simenchelys* has more, most commonly 18–19 in total [8]. Caudal fin ray counts are difficult in fossil eels, but there are no reports of fewer than 16 in total [20].
— Post-temporal absent [8,29]. The post-temporal is present in all Cretaceous forms, although it lacks an ossified lower limb [20,30].
— Epurals absent (figure 4). Only the Cretaceous forms have one or two (*Luenchelys*) epurals [7,20,29,30,47].

The following are derived soft-tissue features of Recent eels and *Protanguilla* unknown in Cretaceous eels:

— Pyloric caeca absent [8].
— Nostrils widely separated, the posterior one just anterior to the orbit [8] (figure 1), except in serrivomerids and nemichthyids.
— Gill membranes united across the isthmus, openings restricted [8] (figure 1).

The following are synapomorphies of Recent eels lacking in *Protanguilla* and Cretaceous eels:

— Premaxilla absent (figure 2). The single tooth-bearing bone that forms the snout in all Recent eels has sometimes been referred to as the premaxillo-ethmovomer, indicating that the ethmovomerine complex (see above) also incorporates the premaxillae dorsally [8,22,30,35,38]. There is no ontogenetic evidence that the premaxillae are fused to the ethmovomer rather than lost [31], nor is there any reason to believe that the incomplete bony tubes that ‘unite posteriorly just in front of the mesethmoid bones’ observed in larval *Anguilla* are rudiments of the premaxillae, as Norman (p. 398 in [35]) suggested. In our opinion, they are more likely to represent rostral ossifications enclosing the ethmoid commissural sensory canal, and further ontogenetic studies are needed. In any case, autogenous but tightly bound premaxillae are present among eels only in *Protanguilla* and the Cretaceous forms.
— Symplectic fused to quadrate (figure 2). Contrary to Robins's statement that ‘there is no ossified symplectic’ in Recent eels (p. 11 in [8]), the symplectic is not lost, but fused with the quadrate during development [31]. The ‘cartilaginous symplectic’ he reported in synaphobranchids [8] is the persistent remnant of the hyosymplectic cartilage between the hyomandibular above and the symplectic below. *Protanguilla* is unique among Recent eels in having a fully autogenous symplectic. In the Cretaceous eels, the symplectic is variously reported as fully autogenous or fused to the quadrate only at its distal tip [7,22,30].
— Metapterygoid absent (figure 2). The suspensorium of all Recent eels lacks a metapterygoid [22,28,30], a cartilage bone primitively and commonly present in teleosts. A separate bone illustrated in that position [26] for *Myroconger* was apparently an artefact owing to breakage. The two small bones described as appearing anterior and lateral to (and eventually fusing with) the hyomandibular in the early development of the ophichthid *Myrophis* [31] are almost certainly membrane components of the hyomandibular, with which their continuity could not be discerned by alizarin staining. *Protanguilla* is unique among Recent eels in having a fully developed and separate metapterygoid, a feature that also characterizes the Cretaceous taxa.
— Upper hypurals fused (figure 4). Cretaceous eels have three autogenous upper hypurals [20,22]. In Recent eels, the upper plate comprises a single plate that may [47] or may not [31] show evidence of originating from two individual hypurals. *Protanguilla* is unique and primitive among them in having the uppermost two hypurals (presumably 3 and 4) free from one another, although the uppermost fuses ontogenetically to the ural centrum.

The following are features of *Protanguilla* in which it is more primitive than all known eels, Recent and Cretaceous:

— Gill rakers present (figure 3). The primitive and most common condition among teleosts, including other elopomorphs, is to have one or (usually) two rows of bony, club-shaped or lathe-like, usually tooth-bearing rakers along the lengths of each hypo-, cerato- and epibranchial, and these toothed rakers are well developed on the gill arches of *Protanguilla*. Such structures are lacking in all other Recent eels and the Cretaceous forms [8,20]. We observed a few smaller and much less extensive flat toothplates near the cerato-epibranchial junction on the first three arches in the synaphobranchid *Synaphobranchus* and the chlopsid *Kaupichthys*, and similar plates may be present in some Cretaceous eels, though this is far from certain.
— Fewer than 90 vertebrae. Most Recent and Cretaceous anguilliforms have vertebral counts between 98 and 200 [25], and counts above 300 are recorded in the nemichthyid *Nemichthys* and the saccopharyngoid *Saccopharynx*. Fewer vertebrae are found only in the Cretaceous *Luenchelys* (90) [22], and the highly specialized saccopharyngoids *Monognathus* and *Cyema*, with as few as 88 [25] and 70 [40], respectively. The low number in *Protanguilla* (79–87) is another primitive feature with respect to anguilliforms in general.
— Pterotic does not approach anterior margin of pterosphenoid, and the latter bone participates in the posterior margin of the orbit (figure 4). All Recent and Cretaceous eels share a specialized configuration in which the elongate pterotic extends near to or beyond the anterior margin of the pterosphenoid, and the basisphenoid articulates dorsally with a ventral flange of the frontal, to exclude the pterosphenoid from the orbital wall [7,20]. The structure sometimes labelled orbitosphenoid in Cretaceous eels [29] is clearly a frontal flange. *Protanguilla* exhibits the primitive and common teleost configuration, in which the pterotic does not reach the anterior margin of the pterosphenoid and the basisphenoid articulates dorsally with the pterosphenoid, which in turn is not excluded from the orbital margin by a frontal flange. A similar condition of the pterotic is found among anguilliforms only in *Coloconger*, wherein the pterosphenoid is partly exposed to the orbit ventrally, though mostly excluded by a large triangular frontal flange (not recognized by Smith [48]) that does not actually articulate directly with the basisphenoid.

The following features are present among Recent eels only in *Protanguilla* and are unknown for Cretaceous forms:

— Pseudobranch present. Although we have not confirmed this histologically, *Protanguilla* bears a corrugated ovoid structure inside the opercular cavity in the usual position of a pseudobranch. A pseudobranch is present in the larvae of Recent eels, but lacking in adults [8].
— Collar-like gill openings (figure 1).

The preponderance of morphological evidence indicates that *Protanguilla* is an anguilliform eel that diverged very early in the evolution of the Anguilliformes and is morphologically more primitive than all living eels. It shares at least 15 characters diagnostic of both Cretaceous and Recent taxa of the order, and seven derived characters of Recent eels lacking in the Cretaceous forms. Most notably, *Protanguilla* differs from all Recent eels in having a premaxilla, metapterygoid, free symplectic and uppermost two hypurals free from one another, all primitive features that also characterize Cretaceous eels, and is more primitive than the latter in having a fully developed set of gill rakers, fewer than 90 vertebrae and a pterosphenoid that forms part of the posterior margin of the orbit.

### (c) Molecular evidence

After confirming the phylogenetic affinity of *Protanguilla* with the true eels within the whole Actinopterygii (electronic supplementary material, figure S1), we performed partitioned ML analysis of the dataset comprising 58 anguilliforms (representing all 19 currently recognized families) plus 11 outgroups. The resulting phylogenies placed *Protanguilla* as sister to three synaphobranchids, the most basal anguilliform clade, with a relatively high bootstrap probability (BP) of 84 per cent (electronic supplementary material, figure S2). Exclusion of the third codon positions from the dataset recovered an identical tree topology regarding the phylogenetic position of *Protanguilla* with a BP of 80 per cent (electronic supplementary material, figure S3).

**Figure 5.**
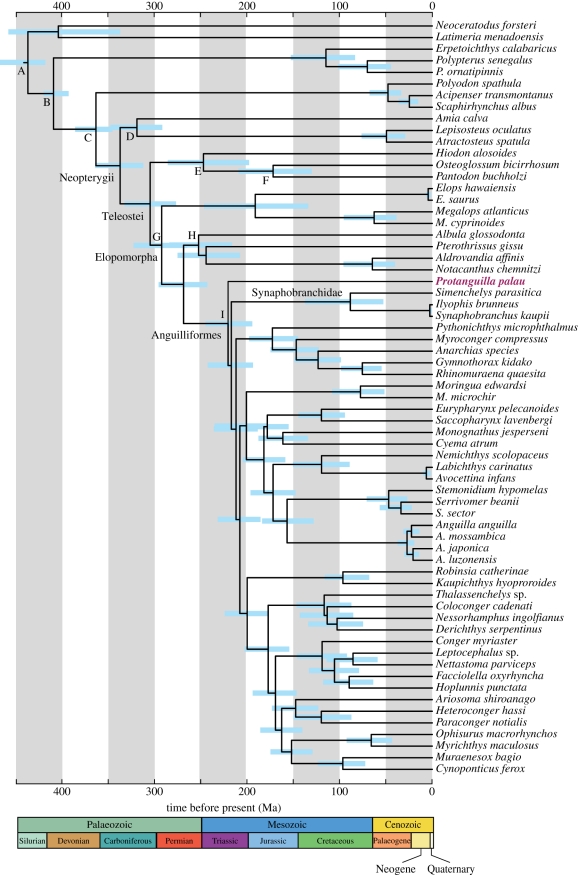
Timetree of anguilliforms and outgroups estimated from relaxed molecular-clock analysis using MCMCTREE implemented in PAML v. 4.4 [18]. One of the constrained, best-scoring ML trees that are congruent with the morphology-based hypothesis in terms of the phylogenetic position of *Protanguilla* was used for divergence time estimation. Nine nodes (A–I) were used for time constraints (for details, see electronic supplementary material, table S3). Horizontal bars indicate 95% credible intervals of divergence time estimates.

Considering a short internal branch from a common ancestor of *Protanguilla* and synaphobranchids in the molecular phylogenies (figure 5; node A in electronic supplementary material, figures S2 and S3) and the robust morphology-based phylogenetic hypothesis, the two BPs (84% and 80% in electronic supplementary material, figures S2 and S3, respectively) are disproportionately high. We therefore evaluated three alternative phylogenetic positions of *Protanguilla* in relation to ‘synaphobranchids’ and ‘other eels’ using the AU test [49]. These are as follows. Tree 1: non-constrained ML tree ((*Protanguilla*, synaphobranchids), other eels) molecular phylogeny (electronic supplementary material, figures S2 and S3). Tree 2: constrained ML tree enforcing monophyly of synaphobranchids + other eels as suggested by the morphological data (*Protanguilla*, (synaphobranchids, other eels)). Tree 3: constrained ML tree enforcing monophyly of non-synaphobranchid eels (synaphobranchids, (*Protanguilla*, other eels)).

AU test shows not only that the latter two hypotheses based on the constrained ML topologies (electronic supplementary material, figures S4 and S5) cannot be rejected (*p* = 0.297 and 0.166 for tree 2, and *p* = 0.404 and 0.361 for tree 3; electronic supplementary material, table S5), but also that the two BPs (84% and 80%) in the non-constrained ML topologies (electronic supplementary material, figures S2 and S3) are somewhat overestimated (*p* = 0.632 and 0.657; electronic supplementary material, table S5). Significantly, *Protanguilla* is placed as the most basal anguilliform taxon in any of the clades (electronic supplementary material, figures S4 and S5), indicating its early divergence. Thus, *Protanguilla* appears to represent an ancient independent evolutionary lineage within anguilliforms, whose placement within the basal anguilliforms is difficult in a molecular phylogenetic context.

### (d) Divergence time

The divergence time analyses are based on the topology that places *Protanguilla* as the sister group of all other eels (tree 2: the hypothesis robustly supported by the morphological evidence). The resultant timetree indicates that *Protanguilla* diverged from other eels during the Triassic–Jurassic boundary around 220 Ma (figure 5; posterior mean 217 Ma with a 95% credible interval between 193 and 243 Ma). Ambiguities in the placement of *Protanguilla* among basal anguilliform lineages have limited impact on the divergence time estimations, with greatly overlapping posterior means and 95 per cent credible intervals of 199 Ma (170–228 Ma) for tree 1 and 203 Ma (181–226 Ma) for tree 3 (electronic supplementary material, table S7). Thus, we consider that *Protanguilla* represents an ancient anguilliform lineage that dates back to the early Mesozoic (around 200 Ma), which is comparable to that of the entire Anguilliformes (posterior means 199–217 Ma; electronic supplementary material, table S7).

### (e) Occurrence

*Protanguilla* is presently known from a single fringing reef cave at 35 m depth in Palau.

As an elopomorph, it almost certainly has a leptocephalus larval form, and letptocephali (particularly those of anguilliforms) are known to have long planktonic durations (2–10 months) [50]. Accordingly, we believe that *Protanguilla* probably has a considerably broader distribution than currently known, even though no leptocephali matching its unique meristic formula (fewer than 90 vertebrae, more than 170 dorsal fin and anal fin rays) have been identified in extensive worldwide larval fish collections. In any case, historically, the *Protanguilla* lineage, estimated to have diverged *ca* 200 Ma, must have been much more widely distributed, because the Palau-Kyushu Ridge formed only around 60–70 Ma [51].
